# Lipopolysaccharide-binding protein (LBP) reverses the amyloid state of fibrin seen in plasma of type 2 diabetics with cardiovascular co-morbidities

**DOI:** 10.1038/s41598-017-09860-4

**Published:** 2017-08-29

**Authors:** Etheresia Pretorius, Sthembile Mbotwe, Douglas B. Kell

**Affiliations:** 1 Department of Physiological Sciences, Stellenbosch University, Stellenbosch Private Bag X1 MATIELAND, 7602 Stellenbosch, South Africa; 2 Department of Physiology, Faculty of Health Sciences, University of Pretoria, Arcadia, 0007 South Africa; 3 School of Chemistry, The University of Manchester, 131 Princess St, MANCHESTER M1 7DN, Lancs, UK; 4 Manchester Institute of Biotechnology, The University of Manchester, 131 Princess St, MANCHESTER M1 7DN, Lancs, UK; 5 Centre for Synthetic Biology of Fine and Speciality Chemicals, The University of Manchester, 131 Princess St, MANCHESTER M1 7DN, Lancs, UK

## Abstract

Type 2 diabetes (T2D) has many cardiovascular complications, including a thrombotic propensity. Many such chronic, inflammatory diseases are accompanied (and may be exacerbated, and possibly even largely caused) by amyloid fibril formation. Recognising that there are few strong genetic associations underpinning T2D, but that amyloidogenesis of amylin is closely involved, we have been seeking to understand what might trigger the disease. Serum levels of bacterial lipopolysaccharide are raised in T2D, and we recently showed that fibrin(ogen) polymerisation during blood clotting can be affected strongly by LPS. The selectivity was indicated by the regularisation of clotting by lipopolysaccharide-binding protein (LBP). Since coagulopathies are a hallmark of T2D, we wondered whether they might too be caused by LPS (and reversed by LBP). We show here, using SEM and confocal microscopy, that platelet-poor-plasma from subjects with T2D had a much greater propensity for hypercoagulability and for amyloidogenesis, and that these could both be reversed by LBP. These data imply that coagulopathies are an important feature of T2D, and may be driven by ‘hidden’ LPS. Given the prevalence of amyloid formation in the sequelae of diabetes, this opens up novel strategies for both the prevention and treatment of T2D.

## Introduction

There is an increasing recognition that many chronic, inflammatory diseases are accompanied (and may be exacerbated, and possibly even largely caused) by amyloid fibril formation^1–6^. An amyloid fibril (protein) is defined as: “a protein that is deposited as insoluble fibrils, mainly in the extracellular spaces of organs and tissues as a result of sequential changes in protein folding that result in a condition known as amyloidosis”^7^. The nature and location of the amyloid typically reflects the disease, such that Parkinson’s is accompanied by amyloid forms of α-synuclein in the substantia nigra pars compacta^8–10^, Alzheimer’s by fibrillar forms of Aβ_1-40/42_ in many parts of the brain^11–13^, and type 2 diabetes – a disease with few genome-wide associations^14^ – by amylin fibrils in the insulin-producing cells of the pancreas^15–25^.

One characteristic of these kinds of fibril is their diameter, which is typically in the range 10–20 nm. By contrast, another type of protein fibre is represented by the fibrin produced during blood clotting. Under normal conditions, the fibres forming the clot have an appearance in a scanning electron microscope like cooked spaghetti, and the fibres have diameters of the order of 80–110 nm^26–30^. We and others have observed that this diameter can be decreased to something closer to 40 nm in various vascular and inflammatory diseases, including stroke^27^ and diabetes^31–33^. This is accompanied by many other changes in overall clot morphology and properties (e.g. for diabetes^31, 33–39^).

Fibrin is produced by the polymerisation of the protein fibrinogen under the action of thrombin, that removes two fibrinopeptides, thereby allowing the fibrinogen to self-assemble via a ‘knobs and holes’ mechanism into fibres (e.g. refs 40–44), but with no particularly marked changes in secondary structure^40, 45–48^. A final, transglutaminase-based crosslinking step^49^ ensures the stability of the clot.

One hallmark of amyloid proteins is the formation of a rich β-sheet structure, perpendicular to the fibres with a characteristic spacing (observable in X-ray reflections) of 4.7–4.8 Å (e.g. refs 4, 50–53, while another is the strong fluorescence observable (when excited ca 440 nm) when thioflavin T is bound to them (e.g. refs 54–59). Normal amyloidosis is rather slow, so needs some kind of ‘trigger’.

Although fibrin too is an insoluble fibre, fibrinogen has generally not been considered as amyloidogenic, nor fibrin as an amyloid protein (although it can become so in the presence of a rare mutation in the fibrinogen a chain^60–63^). However, as part of an extensive study of anomalous blood clotting (e.g. refs 29, 30, 33, 64–68), we recently found^48, 68–70^ that the anomalous clotting was in fact amyloid in nature. The fact that it could be caused by tiny amounts of lipopolysaccharide was seen as consistent with a role for dormant bacteria in the aetiology of such diseases^71–78^. A particularly strong pointer was the fact that the amyloid formation was abolished when the LPS was administered together with a stoichiometric excess of human lipopolysaccharide binding protein (LBP)^68^.

There are also strong indications that both iron dysregulation^64, 79, 80^ and coagulopathies^30, 67^ accompany a variety of inflammatory diseases. Certainly, too, the amount of LPS is raised in diabetics^81–83^, and this can contribute to insulin resistance^84^. Thus, the question then arose as to whether the extent of fibrin-type amyloid in blood varies between type 2 diabetics and suitably matched controls, and whether the removal of any LPS using LBP affected this in any way. The present paper shows that the answers to both these questions are strongly in the affirmative.

## Materials and Methods

### Ethical statement

This study was approved by the Ethical Committee of the University of Pretoria (South Africa): ethics clearance number: 298/2016. A written form of informed consent was obtained from all donors (available on request). The methods were carried out in accordance with the approved guidelines. Blood was collected and methods were carried out in accordance with the relevant guidelines of the ethics committee. We adhered strictly to the Declaration of Helsinki.

### Sample population

26 healthy individuals and 25 type 2 diabetes (T2D) individuals were included in the study. We have discussed the changes in clots of healthy individuals in the presence of LPS and LBP extensively in a recent publication^68^. The present healthy population therefore confirms repeatability of our previous results. Exclusion criteria for the healthy population were known inflammatory conditions such as asthma, human immunodeficiency virus (HIV) or tuberculosis, and risk factors associated with metabolic syndrome (including obesity/high BMI), smoking, and if female, being on contraceptive or hormone replacement treatment. This population did not take any anti-inflammatory medication. As our healthy population were aged between 22 and 91 with BMI < 24.9, we classify them as apparently healthy. We specifically chose not to include controls with increased BMI levels, as literature suggests that an increased BMI is associated with increased levels of inflammation, which may indeed be linked to amyloid formation; furthermore, obesity is also associated with increased endotoxin levels. As there are several levels of increased BMI, from only overweight to morbidly obese, the effect of BMI on clot structure, warrants a separate study.

T2D individuals were voluntarily recruited by their medical practitioner, from a Diabetic Clinic at the Steve Biko academic hospital, Pretoria, South Africa. Demographic data including age, gender, glucose level at time of sample collection, hemoglobin A1c (HbA1c) measures and medication used for typical comorbidities of T2D were recorded. Inclusion criteria consisted of both male and female participants, aged between 40 and 81 (oldest individual), with a diagnosis of T2D for more than 3 months prior to screening and without any signs of infection. Diabetic individuals were diagnosed per the Society for Endocrinology, Metabolism and Diabetes of South Africa (SEMSDA) guidelines. These guidelines follow the American Diabetes Association (ADA) criteria to define T2D. See Table 1 for medication usage. All the T2D individuals had a history of cardiovascular disease, and although a full history of co-morbidities including both macrovascular and microvascular co-morbidities are available in their patient records, we did not record the specific detail for the purposes of this study. All T2D had BMI’s of >25. Exclusion criteria for the T2D were conditions such as previously diagnosed cancer, asthma, human immunodeficiency virus (HIV) or tuberculosis, smoking, and if female, being on contraceptive or hormone replacement treatment. Ethical clearance was obtained from the Human Ethics Committee of the University of Pretoria. Whole blood of the participants was obtained in citrate tubes and platelet poor plasma (PPP) was used for confocal and SEM experiments.

**Table 1:** Demographics for the healthy and the T2D individuals.

HEALTHY INDIVIDUALS (n=26) (BMI: Normal: < 24.9)						
	**Gender**		**Age**			
Median; STD; %	F: 69%; M:31%		59 (± 25)			
**TYPE 2 DIABETES INDIVIDUALS (n=25) (BMI: Overweight: >25)**						
	**Gender**	**Age**	**HbA1c**	**Chol**	**HT**	**AC**
Median; STD; %	F: 56%; M: 44%	61 (± 11)	8.3 (± 2.19)	88%	84%	80%

HbA1c (%): (levels that are >7% are too high); Cholesterol medication (Chol): Simvastatin, Atorvastatin. Hypertension medication (HT): Coversyl, Amlodopin, Carvedilol, Adalat. Anticoagulant medication (AC): Aspirin or Disprin.

### LPS and LPS-binding protein

The LPS used was from *E. coli* O111:B4 (Sigma, L2630). A final LPS exposure concentration of 0.2 ng.L^−1^ and a final LPS-binding protein (LBP) exposure concentration of 2 ng.L^−1^ LBP was used.

### Airyscan and scanning electron microscopy: healthy plasma samples: addition of LPS and LBP with thrombin to plasma

PPP was also incubated for 10 minutes, with either LPS (LPS exposure concentration: 0.2 ng.L^−1^) or LBP (final exposure concentration of 2 ng.L^−1^). Also, samples were prepared where PPP was incubated with LPS (10 minutes), followed by addition of LBP (incubated for 10 minutes). Adding thrombin to either naïve PPP or PPP with the various added products, in the ratio 1:2, created extensive fibrin networks. For Airyscan preparation, before the thrombin addition, we added Thioflavin T (ThT) at a final concentration of 5 μM to 200 μL of various prepared PPP samples (incubated for one minute, and protected from light). For Airyscan samples, a coverslip was placed over the prepared clot, while the clots were washed, fixed in 4% formaldehyde and prepared for SEM according to known SEM preparation methods. Samples were viewed using a Zeiss LSM 510 META confocal microscope with a Plan-Apochromat 163 and 100×/1.4 Oil DIC objective. Excitation was at 488 nm and emitted light was measured at 505–550 nm. A Zeiss cross beam electron microscope was used to study fibrin fibres.

### Airyscan and scanning electron microscopy: type 2 diabetes samples: addition of LBP with thrombin to plasma

Extensive fibrin networks were created by adding thrombin to naïve T2D PPP in the ratio 1:2. PPP was also incubated for 10 minutes with LBP (final exposure concentration of 2 ng.L^−1^). SEM and Airyscan samples were prepared and viewed as for healthy samples.

### Statistical analysis

The non-parametric Mann–Whitney U test (between controls and T2D samples) and the parametric T-test was performed (within samples) using the STATSDIRECT software.

## Results

Table 1 shows demographics for the healthy and the T2D groups, including HbA1c levels of T2D and their medication usage. The healthy individuals were on no medication and did not suffer from any inflammatory condition. Our diabetes population contains patients that are mostly on a plethora of medications for cardiovascular co-morbidities e.g. hyperlipidaemia, hypertension and they have high HbA1c levels. We used the definition and the diagnostic criteria used in defining “diabetes” according to the Society for Endocrinology, Metabolism and Diabetes of South Africa (SEMSDA) guidelines. These guidelines follow the American Diabetes Association (ADA) criteria to define T2D (https://diabetes.medicinematters.com/guidelines/diagnosis/what-is-new-in-the-2017-american-diabetes-association-standards-/11932360).

**Figure 1:**
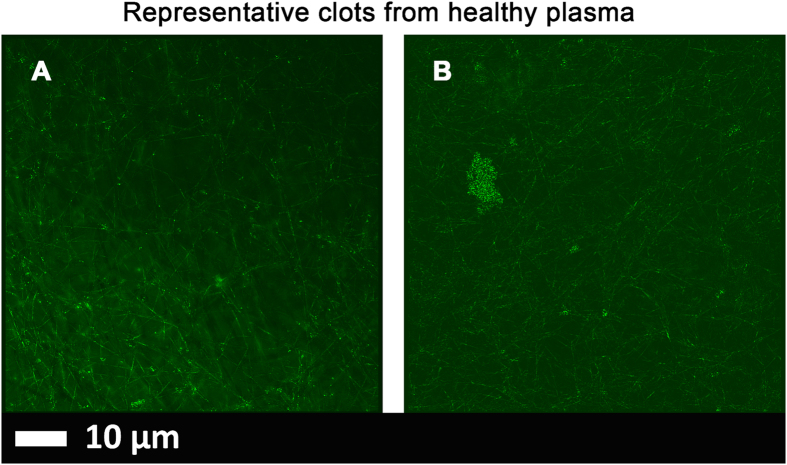
Representative micrographs of PPP clots prepared from blood of two healthy individuals. (**A** and **B**) Micrographs were taken with a Zeiss LSM 510 META confocal microscope with a Plan-Apochromat 63x/1.4 Oil DIC objective. PPP was exposed to thioflavin T (ThT) (5 μM exposure concentration) followed by adding thrombin to create extensive fibrin clots.

**Figure 2:**
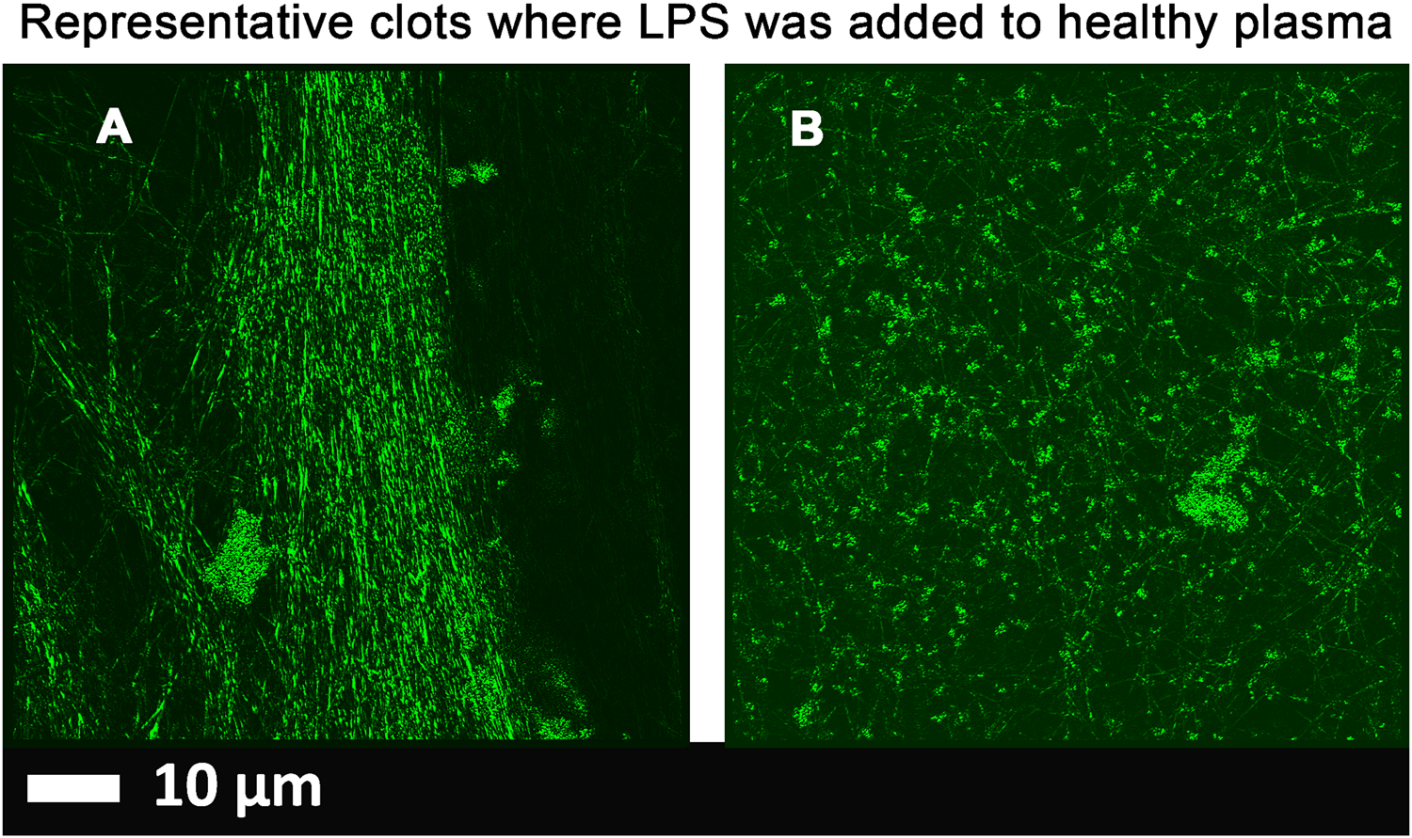
(**A** and **B**) Low magnification micrographs of healthy PPP with added 0.2 ng.L^−1^ LPS Micrographs were taken with a Zeiss LSM 510 META confocal microscope with a Plan-Apochromat 63x/1.4 Oil DIC objective. All PPP was exposed to thioflavin T (ThT) (5 μM) followed by adding thrombin to create extensive fibrin clots. *Areas of increased fluorescence are seen in the great majority of the clot area*.

**Figure 3:**
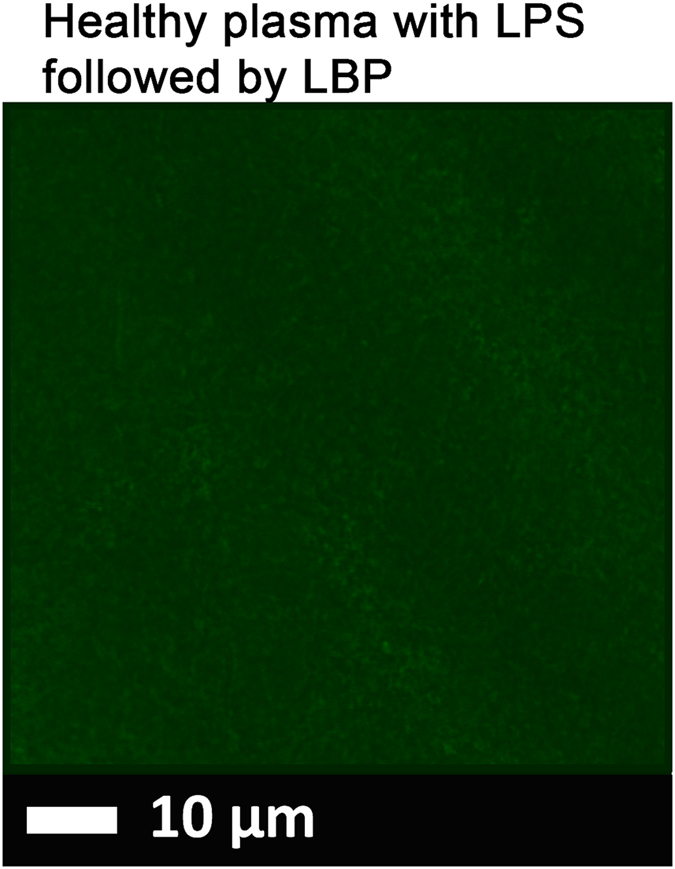
(**A**) A representative micrograph showing healthy PPP with added 0.2 ng.L^−1^ LPS (10 minutes exposure) followed by 2 ng.L^−1^ LPS-binding protein and thrombin. PPP with LPS and LBP was then exposed to thioflavin T (ThT) (5 μM exposure concentration) followed by adding thrombin to create extensive clot. Micrographs were taken with a Zeiss LSM 510 META confocal microscope with a Plan-Apochromatt 63x/1.4 Oil DIC objective. *Nearly no fluorescence is visible*.

**Figure 4:**
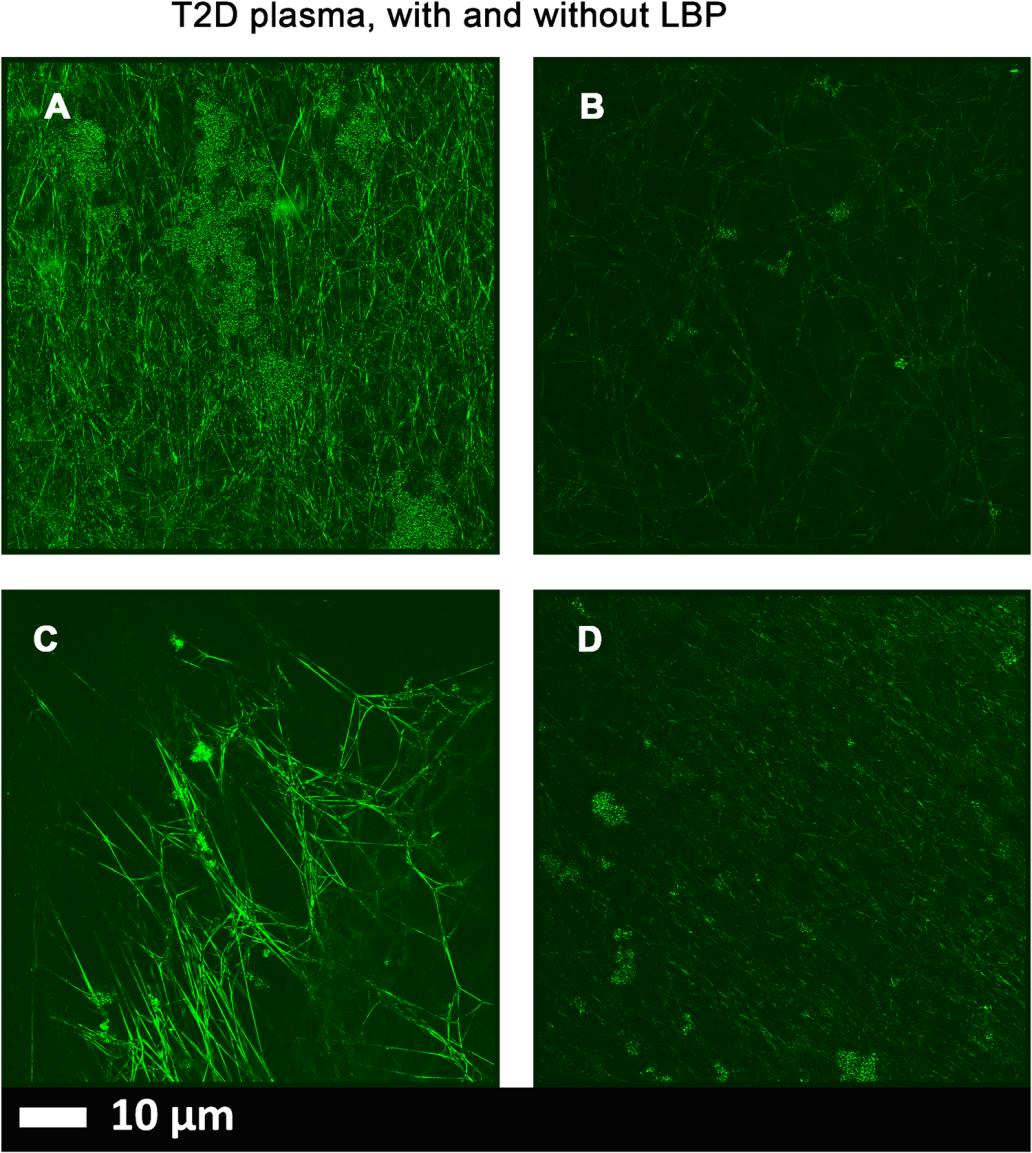
(**A** and **C**) Micrographs of PPP with added thrombin to form extensive fibrin fibres from 2 individuals with type 2 diabetes; (**B** and **D**) PPP from same individuals, but exposed to 2 ng.L^−1^ LPS-binding protein followed by addition of thrombin. Thioflavin T (ThT) (5 μM) was added before thrombin. Micrographs were taken with a Zeiss LSM 510 META confocal microscope with a Plan-Apochromat 63x/1.4 Oil DIC objective. *LBP dramatically reduced the fluorescence seen in samples from patients with T2D*.

Airyscan results are shown in Figs 1, 2, 3 and 4 for the healthy and T2D population.

### Airyscan super-resolution microscopy

Healthy plasma with added LPS and LBP (Figs 1, 2 and 3).

In healthy PPP, in the presence of ThT, little to no fluorescence was noted, although (as in Fig. 1) there were occasional very small patches of fluorescence. However, when LPS had been added to the mixture prior to thrombin, fluorescence was greatly enhanced, suggesting increased binding of ThT to β-sheet-rich areas on the fibrin(ogen) (Fig. 2). Previously, we concluded that LPS binding causes the fibrinogen to polymerise into a form with a greatly increased amount of ß-sheet (in the presence of thrombin), reflecting amyloid formation^68^. This results in a strong fluorescence observable (when excited ca 440 nm) in the presence of ThT (see e.g. refs 56, 57). Here we also showed that even if we add LBP after LPS exposure, less ThT binding (fluorescence) is observed (Fig. 3). This suggests that LBP protects the fibres from LPS damage by binding to the pre-added LPS in the PPP, before clot formation is induced with thrombin.

### Airyscan super-resolution microscopy

Naïve type 2 diabetes with and without added LBP (Fig. 4).

Extensive fluorescence was noted in all our T2D samples after incubation with ThT (examples in Fig. 4A,C). However, when PPPs from T2D patients was incubated with LBP, very little to no fluorescence was observed (Fig. 4B,D). This suggests that the added LBP binds to something, removing its effect, and here we suggest (i) that it is LPS that is present in the naïve T2D, which is known, and (ii) that it is this presence of the LPS that is in part responsible for the anomalous clotting in T2D plasma.

We also looked at the clot structure of plasma from healthy and T2D individuals, with added thrombin, using scanning electron microscopy (SEM). Previously we reported, and we confirm here, that LPS causes healthy plasma to from denser and more hypercoagulable clots (confirmed by using PPP form healthy individuals and purified fibrinogen, using thromboelastography, confocal microscopy, SEM and we also tested whether LPS would bind to fibrinogen directly, using isothermal calorimetry). In this paper, we also showed that added LBP has the capability to reverse the damage of the LPS. We suggested that LPS causes anomalous clotting that is actually amyloid protein^48, 68^.

**Figure 5:**
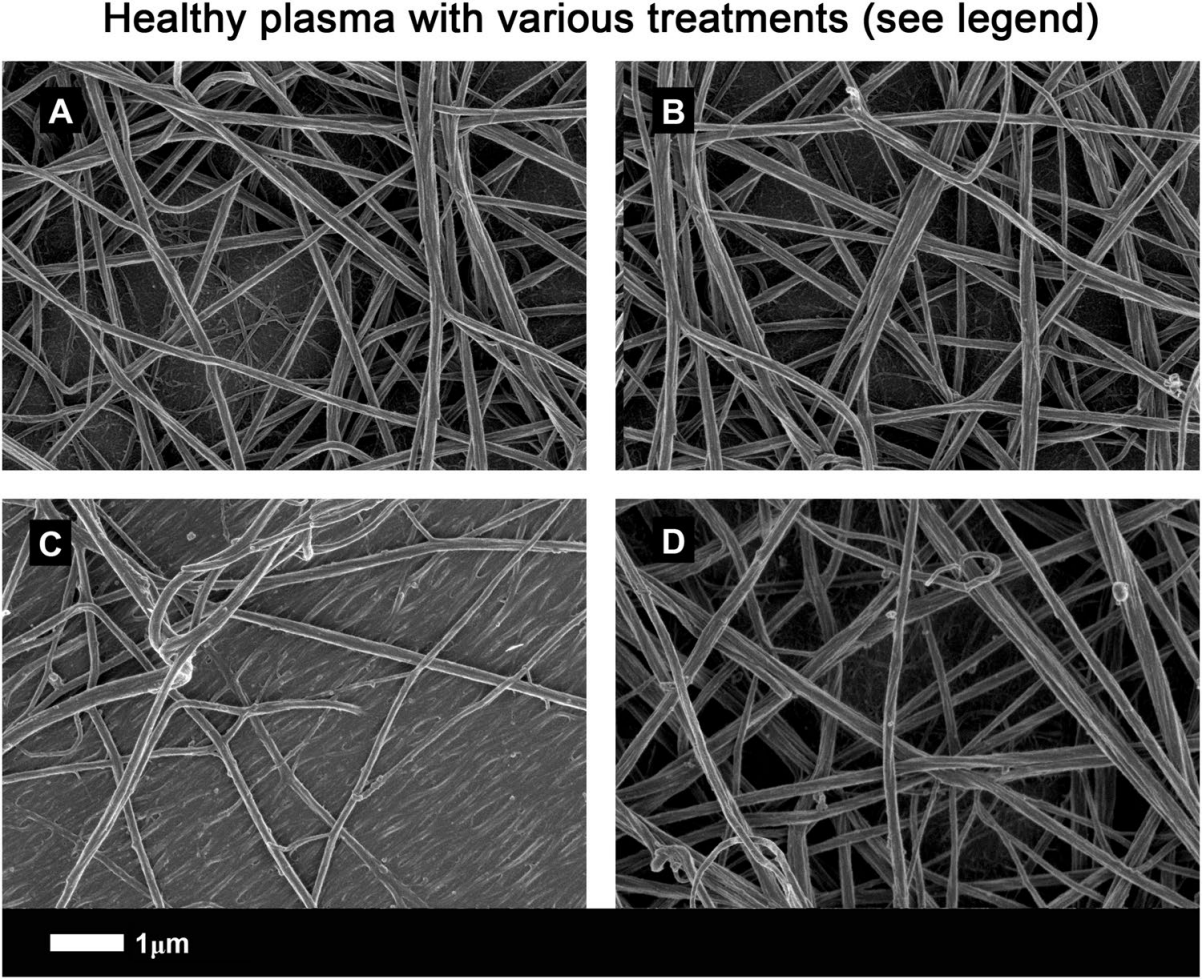
(**A**) Representative clot structure from a healthy individual; (**B**) same PPP sample with added LBP; (**C**) same PPP sample with added LPS; (**D**) same PPP sample with added LPS, followed by LBP. All clots were created by adding thrombin to PPP.

Here, Fig. 5A shows a representative airyscan micrograph of a typical clot from a healthy individual and Fig. 5B shows a clot where LBP was incubated with plasma before clot formation. In our hands, LBP on its own does not change clot structure significantly. Figure 5C shows a clot formed when plasma had been pre-incubated with LPS, while Fig. 5D shows a clot that was formed with plasma, also pre-incubated with LPS, but followed by addition of LBP, and both with added thrombin. Raw data, extensive SOPs for airyscan and SEM, including micrographs can be accessed at https://1drv.ms/f/s!AgoCOmY3bkKHhHp9zfDsIR_0g9_f and on EP’s researchgate profile, https://www.researchgate.net/profile/Etheresia_Pretorius.

**Figure 6:**
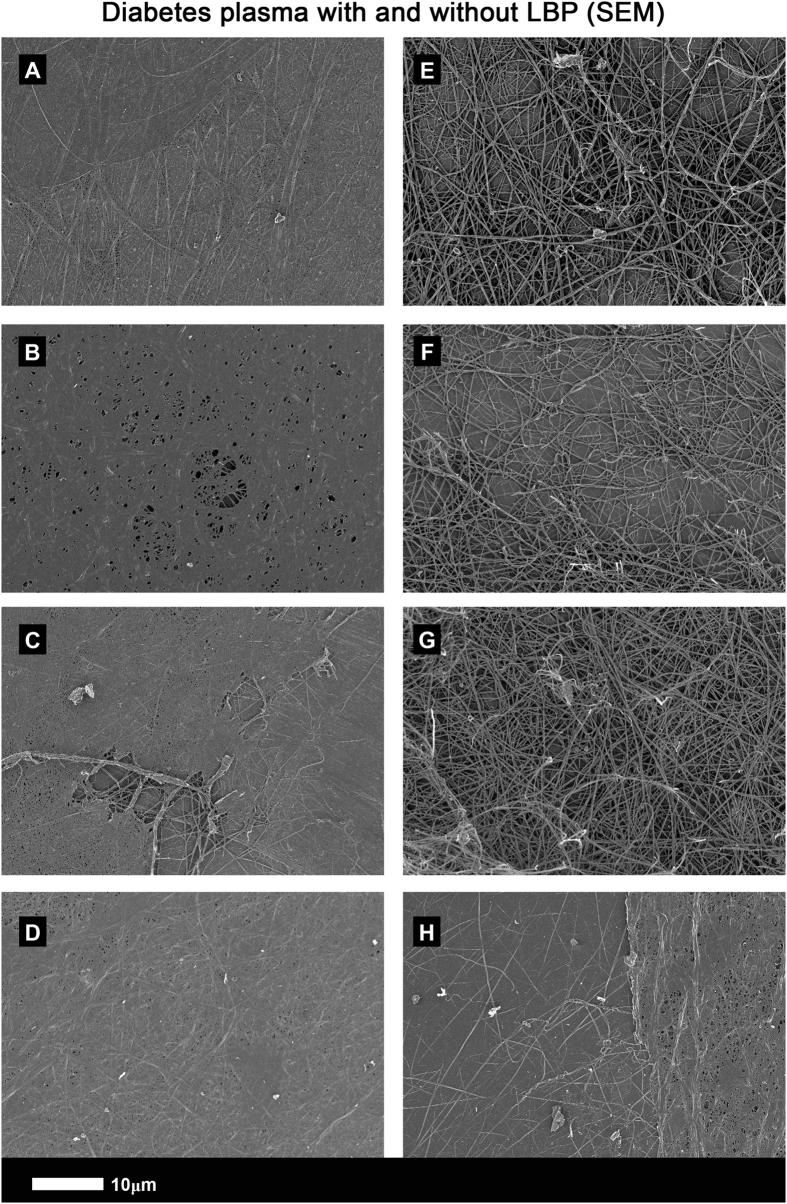
(**A** to **D**) PPP with added thrombin from 4 naïve type 2 diabetes samples (5 k machine magnification); (**E** to **H**) Same samples (5 k machine magnification) with added LBP (incubated for 10 minutes).

**Figure 7:**
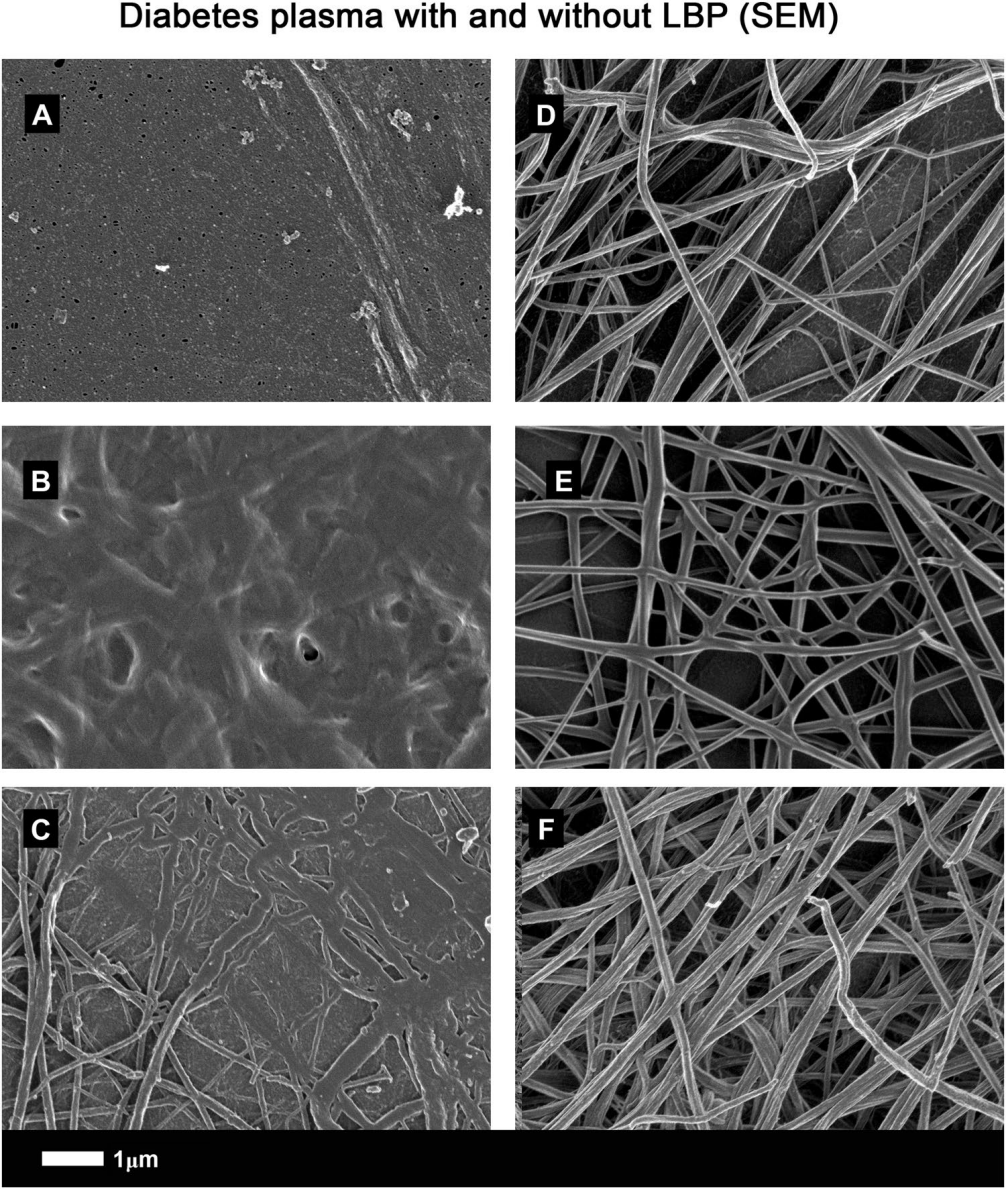
(**A** to **C**) PPP with added thrombin from 3 naïve type 2 diabetes samples (35 k machine magnification); (**D** to **F**) same samples (35 k machine magnification), with added LBP (incubated for 10 minutes).

SEM micrographs show that clots created from T2D PPP (Figs 6 and 7) have a structure similar to those from healthy PPP with added LPS. However, when LBP is added to PPP followed by thrombin, the structure is reverted to that of healthy PPP with added thrombin: see 5 k machine magnification micrographs of clots from T2D (Fig. 6A to D) and with added LBP (Fig. 6E to H). Figure 7A to F show micrographs with higher SEM magnifications (35 k machine magnification). Consequently, in this paper, we show that LBP added to naïve T2D plasma, followed by the addition of thrombin, led to a fibrin fibre structure similar to that of healthy PPP. The fluorescence (Airyscan) results were therefore confirmed by SEM.

**Figure 8:**
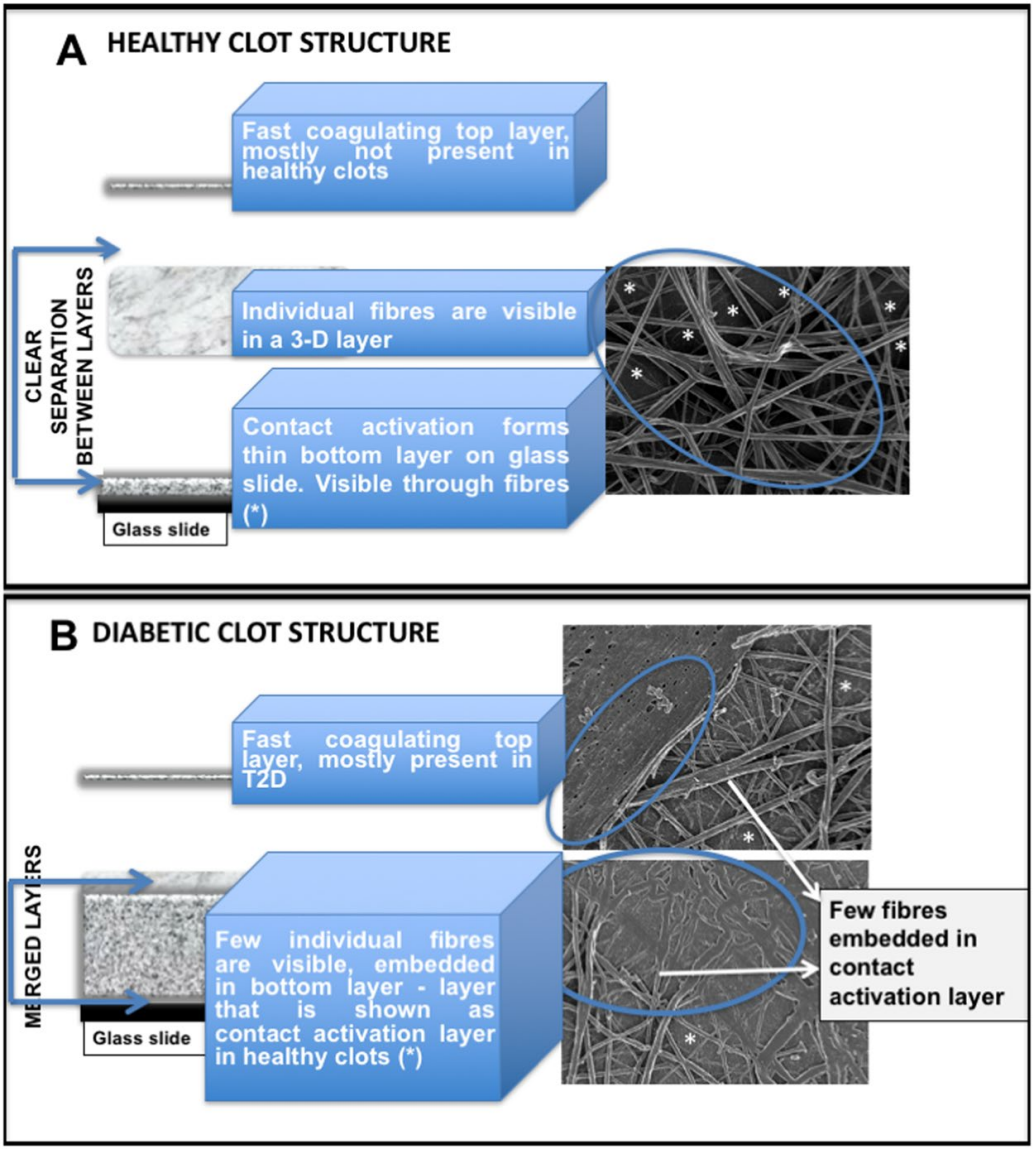
(**A**) Model of a fibrin clot formed from plasma from a healthy individual. (**B**) Model of a clot made using PPP taken from an individual with type 2 diabetes. A typical clot consists of 3 layers, fast coagulating top layer, middle fibrous layer and bottom, contact activation layer. The thickness and presence of these 3 layers vary between clots from healthy individuals and inflammatory clots.

For a schematic model of a ‘healthy’ clot structure (i.e. a clot formed from the PPP of a healthy individual), and that found in T2D, see Fig. 8A,B. A typical clot is created by adding thrombin to PPP, and this is spread out onto a glass coverslip. The coagulum (clot) is then processed by fixation and dehydration. Such processed clots consist of three layers. These layers vary in thickness and structure depending upon whether the plasma was from a healthy individual or an individual with inflammation. A clot from a healthy individual consists of a bottom layer, which is caused by contact activation of the coagulum and the glass substrate. The second layer is the “true” clot, consisting of a fibrin fibre network of elongated fibres. Sometimes, the contact activation layer is visible through the spaghetti-like fibres. Depending on the preparation procedures, a third, top layer may form on the spaghetti-like fibres. This layer typically washes away during the fixing and dehydration process. However, it may sometimes be visible as patches of coagulum, similar to that of the contact activation layer at the bottom (see Fig. 8A). In T2D, the bottom contact activation layer is not easily distinguishable from the middle “true” clot area, and sparse spaghetti-like fibres are fused into the contact activation layer. This fused layer of matted fibrin may consist of thin branching individual fibres, with very small openings between the individual fibres, giving the impression of a matted plate. In previous publications, we have also referred to this complex and hypercoagulable clot structure as “dense matted deposits” (DMDs) (e.g. refs 48, 65, 68, 85). In T2D, a top netted layer is frequently observed. It mostly does not wash away during the various fixing and dehydration steps, and it forms nearly instantaneously when mixing thrombin with plasma to create the clot. This is due to the hypercoagulable nature of the plasma (see Fig. 8B). During confocal clot preparation, a wet clot is studied. The PPP and thrombin are mixed on a microscope slide and covered after 30 second with a coverslip. Although contact activation happens directly on the glass microscope slide, we therefore look at the fibrous part, which is similar to the middle “true clot” layers of the SEM clots in clots from healthy individuals.

**Figure 9:**
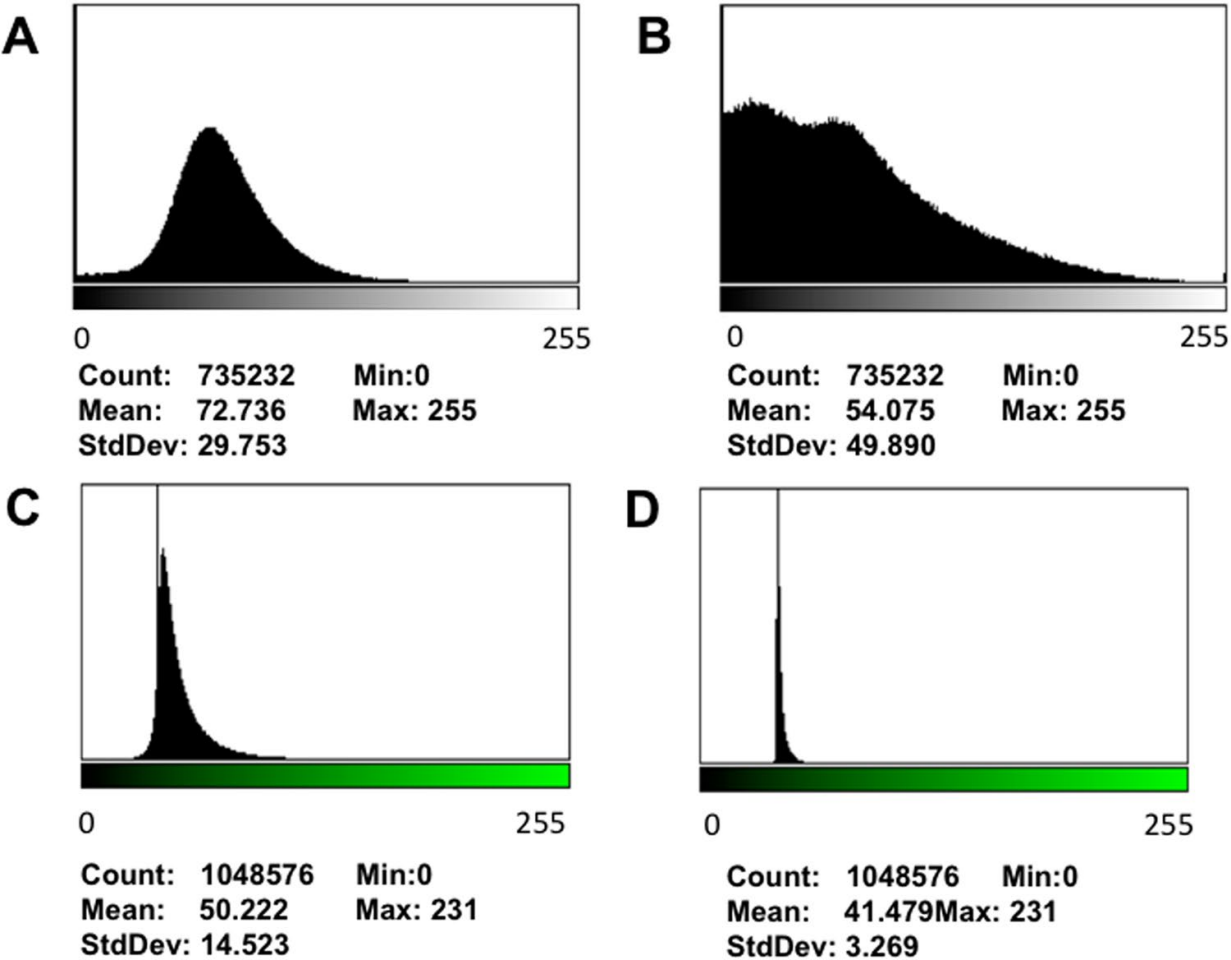
(**A** and **B**) Representative histograms of the 8-bit intensity for a typical SEM clot from PPP of an individual with type 2 diabetes (T2D) (**A**) and after addition of LBP (**B**). (**C** and **D**) Representative histograms of the 8-bit intensity for a typical Airyscan clot from PPP of an individual with T2D (**C**) and after addition of LBP.

**Figure 10:**
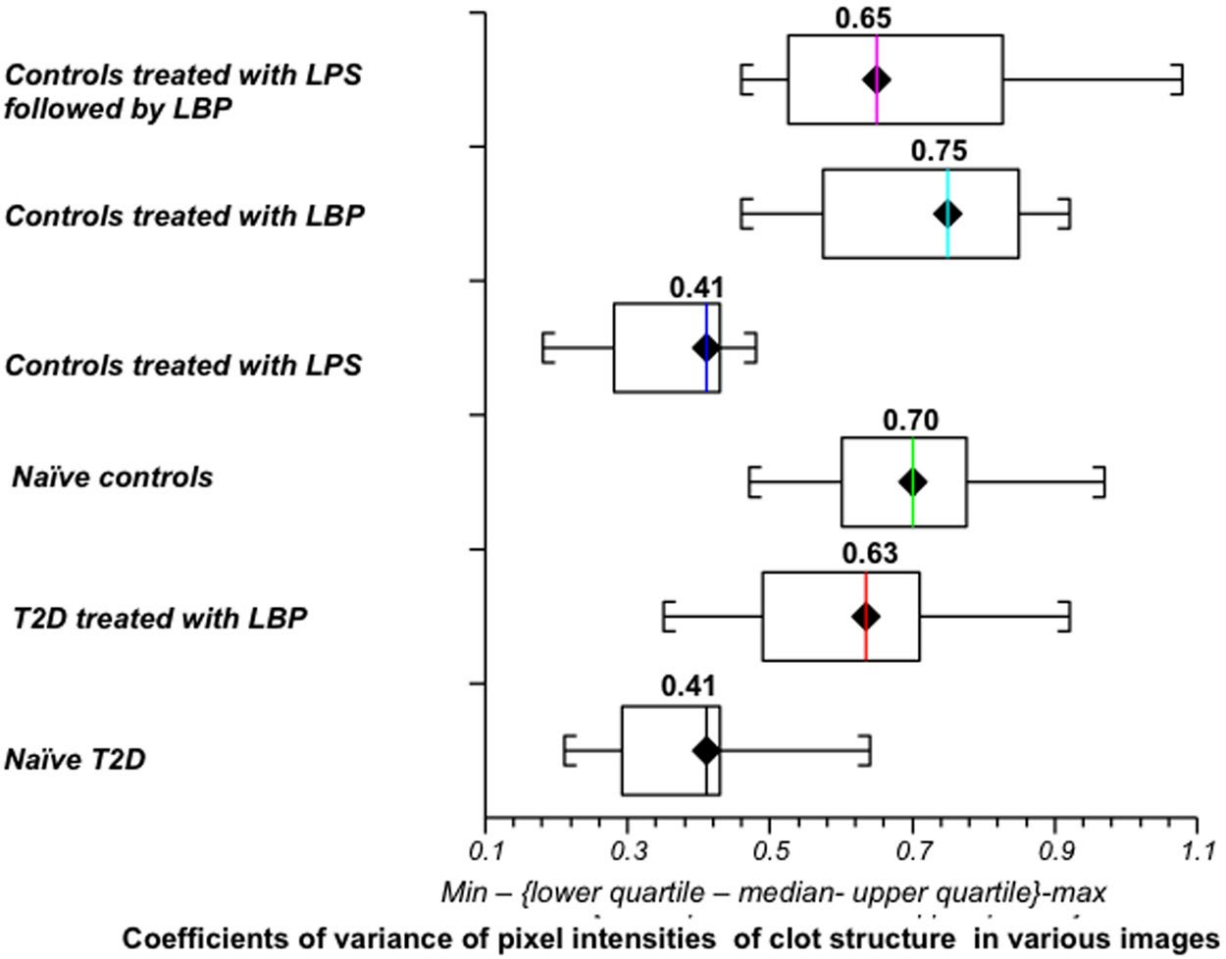
Boxplots of the distribution of the coefficients of variation (CV) of the pixel intensities of the SEM clot images from the different sample classes analysed (median CV for each group is above boxplots). *Note that that the CVs of the clot structures of PPP from naïve T2D and that of healthy individuals with added LPS are both 0.41*.

Although Airyscan and SEM analysis are both typically used only as qualitative methods, we aimed to quantify the changes in clot structure. By inspection (see figures above) the control samples without LPS showed much more variance between light and dark pixels, while the samples forming dense matted deposits were more uniformly grey. It thus seemed logical to use this to seek to quantify and discriminate the images. We therefore used the coefficient of variation (CV) as our metric. Thus, while recognising that the pixel intensity data were not normally distributed, we used ImageJ to calculate the mean and standard deviation of the intensity of the pixels in the images of the clot, using the histogram function, followed by the calculation of the coefficient of variation (i.e. SD/mean) of the intensity of the clot structure. Figure 9A to D show examples of representative histograms of the 8-bit intensity for a typical SEM and confocal clot with and without LBP of a patient with T2D. Within-sample analysis was done with the paired T-test and between-samples analysis was done using the Mann-Whitney test. Table 2 shows statistical analysis of the difference in the coefficients of variation (CV) of the pixel intensities in the images of the different clots studied using SEM and Airyscan technology and Fig. 10 shows boxplots of the data.

**Table 2:** Scanning electron microscopy data for type 2 diabetes and healthy individuals showing the coefficients of variation (CV) of the intensity of the pixels in the clot images and statistical analysis of the difference in the CV of the pixel intensities in the images of different clots studied using scanning electron microscopy and Airyscan technology.

**Type 2 Diabetes Sem Data Coefficient of Variation**			**Healthy Individual SEM data Coefficient of variation**			
	**Naïve T2D**	**T2D treated with LBP**	**Naïve controls**	**Controls treated with LPS**	**Controls treated with LBP**	**Controls treated with LPS followed by LBP**
**MEDIAN AND STD**	0.41 (± 0.10)	0.63 (± 0.14)	0.70 (± 0.12)	0.41(± 0.09)	0.75 (± 0.16)	0.65 (± 0.19)
**Scanning Electron Microscopy**						
**Choice of Samples**	**Test Used**	**P-value**				
Analysis between naïve T2D and naïve controls	Mann-Whitney	***Two sided P < 0.0001***				
Analysis between naïve controls and controls treated with LPS	Paired T-test	***Two sided P < 0.0001***				
Analysis between naïve T2D and T2D treated with LBP	Paired T-test	***Two sided P < 0.0001***				
Analysis between naïve controls and controls treated with LBP	Paired T-test	Two sided P = 0.20				
Analysis between naïve controls and controls treated with LPS followed by LBP	Paired T-test	Two sided P = 0.22				
Analysis between controls treated with LPS and naïve T2D	Mann-Whitney	Two-sided P = 0.82				
Analysis between T2D treated with LBP and naïve controls	Mann-Whitney	Two-sided P = 0.07				
Analysis between T2D treated with LBP and controls treated with LPS followed by LBP	Mann-Whitney	Two sided P = 0.4				
Analysis between controls treated with LBP and T2D treated with LBP	Mann-Whitney	Two sided P = 0.13				
**Airyscan Technology**						
**Choice of Samples**	**Test Used**	**P-value**				
Analysis between naïve T2D and naïve controls	Mann-Whitney	***Two sided P = 0.001***				
Analysis between naïve controls and controls treated with LPS	Paired T-test	***Two sided P = 0.009***				
Analysis between naïve T2D and T2D treated with LBP	Paired T-test	***Two sided P < 0.0001***				
Analysis between naïve controls and controls treated with LPS followed by LBP	Paired T-test	Two sided P = 0.27				
Analysis between controls treated with LPS and naïve T2D	Mann-Whitney	Two sided P = 0.80				
Analysis between T2D treated with LBP and naïve controls	Mann-Whitney	Two sided P = 0.41				
Analysis between T2D treated with LBP and controls treated with LPS followed by LBP	Mann-Whitney	Two sided P = 0.48

As shown in our stored raw data, our controls included 7 young controls (≤30 years). We used the paired t-test to compare SEM data from our younger group to our older group. No significant difference was seen between them (P = 0.1388), suggesting that, in this population, age did not affect clot structure.

## Discussion

Notwithstanding the difficulty of defining its ‘concentration’ in serum^73^, as most of it is bound to macromolecules, it is known that in type 1 diabetes the serum load of endotoxin (LPS) is raised relative to that of controls^86^, and that this is associated with disease progression^87^. There can also be an association between LPS levels in the serum and T2D^81–83^, though the direction of causality (if any) is seemingly not known. It is also known that T2D is accompanied by various kinds of coagulopathies (with both hypercoagulability, e.g. refs 88–95 and hypofibrinolysis^31, 36, 37, 39, 89, 92, 94, 96–101^ being observable.

A question that can arise is why we did not measure the amount of LPS in the plasma. LPS is typically not a molecularly defined substance, and its activity is sometimes reported in ‘endotoxin units’ (EU); this is based on a standard taken from an *E. coli* O55:B5 strain; an approximate relationship is that 1 ng endotoxin ~10 EU^73, 102^. The only assay currently available is the *Limulus* amoebocyte lysate assay; however, its use in the estimation of LPS in blood is not considered especially reliable^103–107^.

Certainly, some of the fibrin(ogen) changes we see in this paper may be due to glycation of fibrinogen by the raised glucose levels^38, 108–112^, as well as the increased levels of fibrinogen itself (e.g. refs 35, 112–115). However, because we had recently shown the extreme sensitivity of the form of the fibrin structure to tiny amounts of (free) LPS, including the formation of amyloid^48, 68^, it was of interest to enquire as to whether diabetic subjects were more likely to produce an amyloid form of clot.

Although we have seen anomalous clotting structures in diabetes before^32, 33, 116^, this is the first time that we have shown them to be amyloid in character. This is entirely consistent with the fact that at least some of the amyloidogenesis must be catalysed by fibrinogen-bound LPS, given too, as we show here, that it could be removed, and the amyloidogenesis largely reversed, by LBP. Interestingly, fibrin deposition has also previously been noted in term placentas from diabetic mothers^117^.

As well as for islets^16, 18–20, 118–122^, amyloid deposition in diabetes is known in kidney^123–125^, as is the deposition of serum amyloid A^126, 127^ (which may have prognostic value^128^). We also note the various comorbidities between T2D and CVD^129–132^; one interpretation is a common cause. In our T2D sample, most of the individuals suffer from hypertension and increased cholesterol and take anticoagulant medication. These co-morbidities are also common in CVD, and T2D is well known as a major cardiovascular risk factor^133^. Both T2D and CVD are known to have vascular pathology^134^, and this is directly associated with coagulation pathology^116^. We also looked to see it there are any relationships been our newly determined blood parameters and e.g. fasting glucose, but we could not discern any.

Endotoxin levels in T2D have previously been found to be, in part, the cause for hypercoagulation and that T2D patients treated with e.g. the anti-glycemic agent, rosiglitazone showed significantly lower endotoxin levels^81^. Our sample did not use this medication. Besides medication for glucose control, our T2D group also includes individuals on medication for dyslipidaemia and hypertension, while some are taking anticoagulants (see Table 1). Anticoagulants (e.g. aspirin/disprin) increase fibrin clot porosity and susceptibility to lysis^135, 136^ (i.e. the exact opposite effect from that noted here with LPS/LBP; Pretorius *et al*. 2013, 2015); they also have an antiplatelet effect^136–139^ and are known to reduce the risk of thrombosis^140–144^. Such products are also inhibitors of both cyclooxygenase COX-1 and COX-2^145^. We could not find evidence in the literature that LBP acts as an anti-coagulant.

## Concluding remarks

T2D is accompanied by long-term inflammation, and this inflammation is mediated in part by increased fibrinogen levels, as well as a changed cytokine profile that is driven, at least in part, by dysregulated glucose and insulin function. The origin of this inflammation is mostly unclear and remains unresolved in diabetes. It is known that gut dysbioses and atopobioses^71^ (colloquially referred to as ‘leaky gut’) are a well-known contributor to the pathogenesis of many metabolic diseases, including obesity^146^, T1D^147, 148^, T2D^146, 149, 150^, and CVD^151^. We have previously suggested that there is a fundamental link between gut dysbioses, the presence of a (dormant) blood microbiome and the presence of the highly inflammatory LPS^71–73^. As discussed in the introduction and shown recently, LPS is known to cause hypercoagulability and anomalous blood clotting, even at very low concentrations^48, 68^. We have also shown that LPS may directly bind to fibrinogen monomers^68^. Anomalous clotting is well-known in T2D^31, 33–39^, and here we show that LBP added to PPP from individuals with T2D changes fibrin structure to resemble that of healthy fibrin. We confirmed our results with healthy PPP, adding LPS and mopping it up again with added LBP. It is known that LBP is an acutely induced plasma protein that binds avidly to LPS aggregates, and delivers them to CD14^73, 152, 153^. Our results therefore suggest that, at least in part, the anomalous blood clotting seen in T2D is due to the presence of the potent inflammagen LPS, that can be removed by LBP. A future study could additionally investigate the effect of glucose addition to healthy PPP, with and without the addition of LPS. This will give insights regarding amyloidogenesis and the effects of glucose on such a process. It now seems no coincidence that most of the more serious sequelae of diabetes are accompanied by amyloid formation (e.g. retinopathy^154–156^, nephropathy^123, 157–160^, neuropathy^161^, and cardiovascular problems^162, 163^). It is well-known that all of these macro- and microvascular complications that form part of the T2D comorbidities cause hypercoagulation and are associated with an upregulated inflammatory profile. A follow-up study should investigate the specific influences of comorbidities like a history of macrovascular (cardiovascular diseases such as coronary artery disease, ischemic stroke, and peripheral artery disease), and microvascular (retinopathy, nephropathy, and neuropathy). This will give further insight with regards to the exact role that these comorbidities play in hypercoagulation.

Thus, the recognition from the present work that amyloid formation is a strong function of the presence of cryptic LPS in type 2 diabetes might have important applications in both the treatment of inflammation in T2D, and the progression of the condition generally. The fundamental question that now remains is how to translate these results into clinical practice.
